# Efficacy of non-artemisinin- and artemisinin-based combination therapies for uncomplicated falciparum malaria in Cameroon

**DOI:** 10.1186/1475-2875-9-56

**Published:** 2010-02-19

**Authors:** Solange Youdom Whegang, Rachida Tahar, Vincent Ngane Foumane, Georges Soula, Henri Gwét, Jean-Christophe Thalabard, Leonardo K Basco

**Affiliations:** 1 Unité de Recherche 77 Paludologie Afro-tropicale, Institut de Recherche pour le Développement (IRD) and Laboratoire de Recherche sur le Paludisme, Organisation de Coordination pour la lutte contre les Endémies en Afrique Centrale (OCEAC), BP 288, Yaoundé, Cameroon; 2 Université de Yaoundé I, Ecole Nationale Supérieure Polytechnique, Département de Mathématiques et Sciences Physiques, BP 8390, Yaoundé, Cameroon; 3 Université Paris Descartes, Laboratoire de Mathématiques Appliquées Paris 5 (MAP5), Unité Mixte de Recherche 8145, Centre National de la Recherche Scientifique, 45 rue des Saints Pères, 75006 Paris, France; 4 Centre de Formation et de Recherche en Médecine et Santé Tropicale, Faculté de Médecine, 13916 Marseille, France

## Abstract

**Background:**

The use of drug combinations, including non-artemisinin-based and artemisinin-based combination therapy (ACT), is a novel strategy that enhances therapeutic efficacy and delays the emergence of multidrug-resistant *Plasmodium falciparum*. Its use is strongly recommended in most sub-Saharan African countries, namely Cameroon, where resistance to chloroquine is widespread and antifolate resistance is emerging.

**Methods:**

Studies were conducted in Cameroonian children with acute uncomplicated *P. falciparum *malaria according to the standard World Health Organization protocol at four sentinel sites between 2003 and 2007. A total of 1,401 children were enrolled, of whom 1,337 were assigned to randomized studies and 64 were included in a single non-randomized study. The proportions of adequate clinical and parasitological response (PCR-uncorrected on day 14 and PCR-corrected on day 28) were the primary endpoints to evaluate treatment efficacy on day 14 and day 28. The relative effectiveness of drug combinations was compared by a multi-treatment Bayesian random-effect meta-analysis.

**Findings:**

The results based on the meta-analysis suggested that artesunate-amodiaquine (AS-AQ) is as effective as other drugs (artesunate-sulphadoxine-pyrimethamine [AS-SP], artesunate-chlorproguanil-dapsone [AS-CD], artesunate-mefloquine [AS-MQ], dihydroartemisinin-piperaquine [DH-PP], artemether-lumefantrine [AM-LM], amodiaquine, and amodiaquine-sulphadoxine-pyrimethamine [AQ-SP]). AM-LM appeared to be the most effective with no treatment failure due to recrudescence, closely followed by DH-PP.

**Conclusion:**

Although AM-LM requires six doses, rather than three doses for other artemisinin-based combinations, it has potential advantages over other forms of ACT. Further studies are needed to evaluate the clinical efficacy and tolerance of these combinations in different epidemiological context.

## Background

Chloroquine-resistant *Plasmodium falciparum *is now widespread in Africa, and antifolate-resistant *P. falciparum *is emerging in some regions in Africa [1]. In Cameroon, chloroquine is not effective, and its importation into the country has been officially stopped in 2002. Amodiaquine and sulphadoxine-pyrimethamine were recommended for the first- and second-line treatment of *P. falciparum *infections, respectively, between 2002 and 2004.

To overcome drug-resistant malaria, malaria experts advocate the use of combination therapy [2,3]. The most commonly recommended combinations for Africa include non-artemisinin-based combinations, such as amodiaquine-sulphadoxine-pyrimethamine (AQ-SP), and artemisinin-based combinations, such as artesunate-amodiaquine (AS-AQ), artesunate-sulphadoxine-pyrimethamine (AS-SP), and artemether-lumefantrine (AM-LM). Other forms of artemisinin-based combinations include artesunate-mefloquine (AS-MQ), dihydroartemisinin-piperaquine (DH-PP), artesunate-chlorproguanil-dapsone (AS-CD), artesunate-pyronaridine, and artesunate-atovaquone-proguanil.

Cameroonian health authorities recommend AS-AQ for the treatment of uncomplicated malaria since 2004. AM-LM is an alternative therapy in Cameroon since 2006. In the previous studies, the results of the nationwide evaluation of the current therapeutic efficacy of monotherapies (chloroquine, amodiaquine, and sulphadoxine-pyrimethamine) were presented [4]. As part of the national surveillance programme of drug-resistant malaria and follow-up studies on combination therapies initiated in 2001, the present series of subtrials presents the current efficacy of AQ-SP, AS-AQ, AS-SP, AS-MQ, AM-LM, AS-CD, and DH-PP [5]. The aim of this series of subtrials was to constitute a database of anti-malarial drug efficacy. These findings provide a rational basis to consolidate the on-going implementation of ACT throughout the country and provide baseline data for possible adjustment and modifications in the national anti-malarial drug policy in the future.

## Methods

### Patients

Clinical studies were conducted at four different urban centres situated in different geographic area in Cameroon. Malaria transmission is intense and continuous throughout the year in the country, except for the northern (Garoua) and far-northern provinces (Maroua), where transmission is low and seasonal. Children were enrolled after free and informed consent of the parents and/or legal guardians if the following inclusion criteria were met: age ≤ five years of age, fever at the time of consultation, parasite density ≥ 2,000 asexual *P. falciparum *parasites/μL of blood, without other *Plasmodium *species [6]. As recommended by the standardized World Health Organization (WHO) protocol for areas of low transmission, the inclusion criteria were extended to children up to nine years of age in Garoua and children of all ages and adults in Maroua. Patients with symptoms associated with concomitant infectious diseases, severe malnutrition, or any danger signs as defined by the WHO were excluded. Study arms were calibrated based on WHO criteria [6]. Each substudy was an open-label trial using drugs both commonly available and commonly used in the selected health care centres. The studies were approved by the Cameroonian National Ethics Committee and the Cameroonian Ministry of Public Health before the initiation of the first campaign in 1995 and amended in 1997 (Number UYI/FMSB/DEPT/HEMAT/L.No 35/95). The study protocol was initiated in 2003 and extended up to 2007, with yearly campaigns.

### Treatment and follow-up

Patients were randomized to two or three treatment groups, with the exception of the study conducted in Maroua where only AS-AQ combination was evaluated. Separate concealed-random list based on random number tables was prepared for each trial by the principal investigator. Patients were consecutively allocated by the local investigator according to the corresponding list. Amodiaquine (AQ) was administered at a standard dose of 10 mg base/kg body weight on days 0, 1, and 2. Sulphadoxine-pyrimethamine (SP; 25 mg/kg body weight sulphadoxine and 1.25 mg/kg body weight pyrimethamine) was administered in a single dose. The dosage of AQ-SP was the same as that of monotherapies. The first doses of AQ and SP were administered simultaneously on day 0, followed by AQ alone on days 1 and 2.

AS was administered at a total dose of 12 mg/kg body weight (4 mg/kg body weight on days 0, 1, and 2) for all ACTs containing AS. The following dosages of ACT were administered: AS-AQ (AS, 4 mg/kg/day and AQ, 10 mg/kg/day) on days 0, 1, and 2; AS-SP, (SP on day 0); AS-MQ (MQ, 15 mg/kg on day 1 and 10 mg/kg on day 2); and DH-PP (Duo-Cotecxin^®^) 6.4 mg/kg body weight of DH and 51.2 mg/kg body weight of piperaquine in 3 divided daily doses. Six doses of AM-LM (Coartem^®^) were administered as recommended by the manufacturer. For the AS-CD combination, the dose of chlorproguanil-dapsone (Lapdap^®^) was given once daily for three days, as recommended by the manufacturer. Paracetamol (30 mg/kg body weight/day) was administered to all patients.

Patients included in the respective AQ, SP, AQ-SP arms of the 2003-subtrial were followed on days 1, 2, 3, 7, and 14, as recommended by the 1996 WHO protocol [7]. All patients included in the subtrials after 2003 were followed on days 1, 2, 3, 7, 14, 21, and 28 (also day 42 for patients assigned to AS-MQ group), as recommended in the WHO protocol modified in 2003 [6]. Haematocrit measurement was repeated on day 14. Each dose of anti-malarial drugs was administered under supervision during the visits. Patients who failed to respond to the assigned drug were treated with oral quinine (25 mg/kg body weight/day for 5 days), artesunate-amodiaquine, or artemether-lumefantrine. The primary outcome was an adequate clinical and parasitological response (ACPR) on day 28 [6]. For comparison, ACPR on day 14 was also considered.

### Polymerase chain reaction (PCR)

Fingerprick capillary blood was collected for blood smear and DNA analysis at the time of treatment or parasitological failure occurring on day 7 or after. The polymorphic merozoite surface antigen-1 (*msa*-1), merozoite surface antigen-1 (*msa*-2), and glutamine-rich protein (*glurp*) genes of the pre-treatment and recrudescent samples were amplified, as recommended by a group of malaria experts [8]. PCR products of pre-treatment and post-treatment samples were analysed by agarose gel electrophoresis.

### Statistical analyses

Both intention-to-treat and per protocol analyses on the percentage of ACPR on day 14 and 28 were performed. Proportions of late failure occurring after day 14 were corrected for re-infection by comparing the PCR products of pre-treatment and post-treatment isolates. The calculations were based on both PCR-uncorrected and PCR-corrected proportions of ACPR for the 28-day studies.

For the 14-day follow-up study (in 2003), significant difference between AQ, SP, AQ-SP arms was tested using ANOVA for the binary variable ACPR 1/treatment failure 0. The test of the efficacy trend of AQ-SP between 2003 and 2006 was performed by comparing the rate of ACPR in 2003 to the rate in 2006 using the odds ratio (OR) on day 14.

For each 28-day follow-up study (2005-2007), the ORs and 95% confidence intervals were calculated. The Yusuf and Peto method was used for the 2006-study (AS-AQ versus AM-LM) as the AM-LM arm showed 100% ACPR patients after PCR adjustment [9]. On days 2, 3, 14 and 28, we used a logistic regression model to compare other forms of ACT, with AQ-SP as the reference treatment. Time to parasite clearance was compared using the log-rank test.

Unlike the classical random-effect meta-analysis, where there is the same reference treatment or placebo across the trials, a pooled effect and summary OR versus a reference treatment could not be directly estimated since treatments were not the same from one study to the other [10]. As the same treatment was repeatedly found in some of the arms among the different trials, a general Bayesian model referred to as a multi-treatment random-effect meta-analysis was used, taking into account the heterogeneity between studies and regrouping these data to compare treatments. The model used was an extension of the one proposed for individual patient data [11].

The treatment response per subject was viewed as a binary variable, i.e. 1 for ACPR and 0 for failure. Data were agglomerated on day 14 for all studies, and on day 28 based on both PCR- uncorrected and corrected results when the follow-up reached 28 days or more. The model estimated i) posterior OR for each treatment compared to the AS-AQ treatment, ii) the variability among subjects within sub-trials and iii) the variability among treatment groups, starting with reasonable prior distribution for each parameter.

Data were analysed using the statistical software R [12]. For the Bayesian random-effect meta-analysis, the WinBugs14 software was used [13].

## Results

### Study population

**Figure 1:**
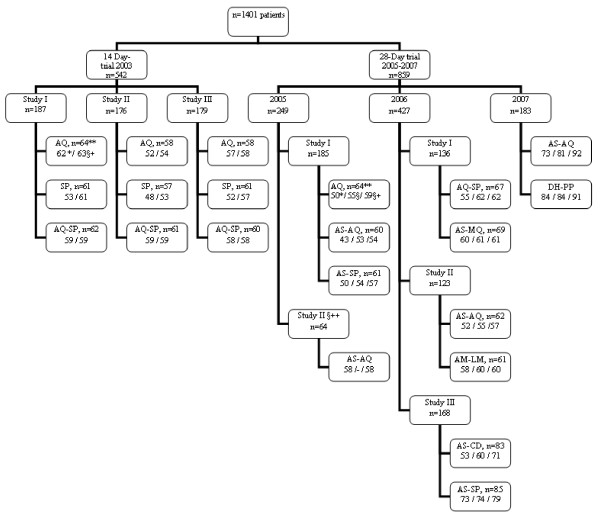
**CONSORT flow diagram**. ** The number of includedpatient in the corresponding treatment group. *The number of observedACPR without PCR correction. § The number of ACPR corrected by PCR. §+: The total number of patient followed (number of observed ACPR + number of failures). The number of patient either lost to follow-up or excluded is obtained by subtracting §+ from **. §++ a non randomized study of AS-AQ.

A total of 1,401 patients were enrolled in our series of studies (1,337 in randomized studies and 64 in a single non-randomized AS-AQ study in Maroua). All analysed data are presented in a CONSORT format in Fig. 1[14]. Patients included in 2003 (n = 542) were followed up until day 14. Patients included in 2005-2007 (n = 859) were followed up until day 28 (until day 42 for AS-MQ). The clinical and parasitological characteristics are presented in Additional File 1. In 2003, 519 patients completed the visit on day 14, and 23 (4.2%) were either lost to follow-up or excluded. In the 28-day trial, 734 patients completed the visits on day 28; and 61 (7.6%) were either lost to follow-up or excluded. The overall mean haematocrit increased from 18.5 ± 1.7% on day 0 to 31.3 ± 5.6% on day 14, i.e. by 12.8% (95% CI, 11.2-14.4%; *P *< 0.05). Even among patients with relatively high initial parasitaemia (> 200,000 asexual parasites/μL of blood; n = 32), the mean pre-treatment haematocrit (28.5 ± 5.4%) increased to 34.6 ± 4.0% on day 14, i.e. increase by 6.2% (95% CI, 4.3-8.0%; *P *< 0.05), attesting the general benefit of an effective combination therapy.

### Efficacy of AQ, SP, AQ-SP

**Figure 2:**
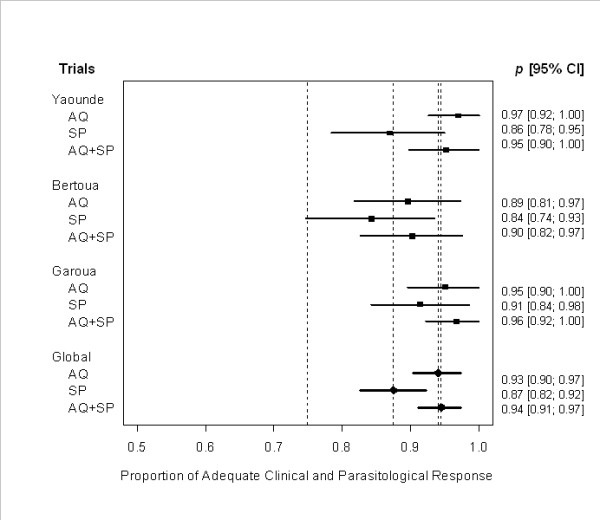
**Comparison of the efficacy of AQ monotherapy, SP monotherapy, and AQ-SP combination in 3 sites during the 14-day follow-up period in 2003**. Individual success rates (PCR uncorrected) are plotted. The horizontal line represents 95% confidence interval (CI) of each estimated proportion p, which is based on asymptotic normality. Black squares on each line denote the estimated proportion of adequate clinical and parasitological response (ACPR). The first dotted vertical line to the left corresponds to 75% of ACPR under which the treatment is considered as ineffective. The last 3 rows and their corresponding vertical lines refer to the global effect observed for sulphadoxine-pyrimethamine (SP; 88% ACPR), amodiaquine (AQ; 93% ACPR), and amodiaquine-sulphadoxine-pyrimethamine (AQ-SP; 94% ACPR), respectively.

With the AQ-SP treatment, the overall cure rate, i.e. ACPR, was 93.0% on day 14 and 78% on day 28 before PCR correction and 91% after PCR correction (Figure 2). There was no indication of change in the efficacy of AQ monotherapy between 2003 and 2005 (OR = 1.61, 95% CI 0.6-4.54) on day 14. SP was less effective than AQ-SP (OR = 0.33; 95% CI, 0.14-0.77; *P *= 0.01), with an overall cure rate of 87% (95% CI, 0.82-0.92) on day 14. In 2003, the efficacy of AQ-SP was not statistically different from AQ monotherapy (*P *> 0.05). There was no significant difference in the efficacy of AQ-SP between 2003 (145/156 or 93%) and 2006 (64/67 or 96%) on day 14 (OR = 0.62; 95% CI, 0.16-2.3).

### Efficacy within the 28-day trial

From 2005 to 2007, the efficacy of artemisinin derivatives combined with a partner drug was assessed on day 28. The treatment outcomes of combination therapies, before and after PCR adjustment of the number of ACPR, are summarized in Tables 1 and 2. In 2005, for AQ, AS-AQ and AS-SP, based on the intention-to-treat (ITT) analysis, the rates of success were, for the uncorrected response, 78.1%, 71.6%, and 82%, respectively and after PCR correction, 86%, 88.3% and 88.5%, respectively. There was no significant difference among the 3 treatments. Between AS-AQ and AM-LM, the success rate based on the PCR-uncorrected proportions of ACPR on day 28 was not significantly different (ITT: OR = 0.26; 95% CI, 0.07-1.03). After PCR correction, the 28-day cure rates were 88.7% and 98.3% for AS-AQ and AM-LM, respectively (ITT: OR = 0.13; 95% CI, 0.01-1.10). Moreover, the time to obtain parasitological clearance was similar in the two groups (*P *= 0.13). AS-AQ combination was less effective (PCR-uncorrected ACPR, 79.3%) than DH-PP (PCR-uncorrected ACPR, 92.3%) (ITT OR = 0.32; 95% CI, 0.12-0.80; per protocol [PP] OR = 0.12; 95% CI, 0.02-0.52). After PCR adjustment, the cure rates on day 28 were 92.7% and 88.0% for DH-PP and AS-AQ, respectively (ITT OR = 0.61; 95% CI, 0.22-1.66). Parasite clearance time was longer with DH-PP than AS-AQ (*P *< 0.05). Based on the ITT analysis, the AS-CD combination was less effective (PCR-uncorrected ACPR, 63.8%) than AS-SP (PCR-uncorrected ACPR, 85.9%) (OR = 0.30; 95% CI, 0.13-0.62). After PCR adjustment, the cure rates on day 28 were 91.7% and 76% for AS-SP and AS-CD, respectively (OR = 0.40; 95% CI, 0.17-0.85).

**Table 1:** Distribution of the responses in the 28-day trial

Year	Treatment	Number included	Parasite clearance on day 3 (ITT) (%)	Observed ACPR	Lost to follow-up and excluded	Reinfection	ITT^1^	PP^1^
2005	AQ	64	87.5	50	5	5	50/64 (55/64)	50/59 (55/59)
	AS-AQ	60	100	43	6	10	43/60 (53/60)	43/54 (53/54)
	AS-SP	61	96.8	50	4	4	50/61 (54/61)	50/57 (54/57)
								
2006a	AQ-SP	67	86.6	55	5	7	55/67 (62/67)	55/62 (62/62)
	AS-MQ	69	95.7	60	8	1	60/69 (61/69)	60/61 (61/61)
								
2006b	AS-AQ	62	98.4	52	5	3	52/62 (55/62)	52/57 (55/57)
	AM-LM	61	100	58	1	2	58/61 (60/61)	58/60 (60/60)
								
2006c	AS-CD	83	97.6	53	12	7	53/83 (60/83)	53/71 (60/71)
	AS-SP	85	100	73	6	1	73/85 (74/85)	73/79 (74/79)
								
2007	AS-AQ	92	99	73	4	8	73/92 (81/92)	73/88 (81/88)
	DH-PP	91	100	84	5	0	84/91 (84/91)	84/86 (84/86)

^1^Values in parentheses denote the observed proportions of ACPR after PCR correction in each treatment group. No recrudescence was observed with the AM-LM combination, and no re-infection was observed with DH-PP. ITT, intention-to-treat; PP, per protocol.

**Table 2:** Outcome of intent-to-treat and per-protocol analyses on day 28 with PCR distinction between recrudescence and reinfection.

Comparator^1^	Treatment	PCR-uncorrected outcome		PCR-corrected outcom	
		
		OR (ITT)	OR (PP)	OR (ITT)	OR (PP)
AS-AQ	AQ	0.71 (0.31-1.60)	0.70 (0.26-1.85)	1.24 (0.43-3.56)	3.85 (0.41-35.6)
AS-AQ	AS-SP	0.60 (0.23-1.31)	0.55 (0.20-1.53)	0.98 (0.32-3.00)	2.94 (0.30-29.2)
AQ-SP	AS-MQ	0.68 (0.26-1.75)	0.13 (0.01-1.10)	1.62 (0.50-5.24)	- -
AS-AQ	AM-LM	0.26 (0.07-1.03)	0.36 (0.06-1.92)	0.13 (0.01-1.10)	0.12 (0-2.04)
AS-CD	AS-SP	0.30* (0.13-0.62)	0.24* (0.09-0.65)	0.39* (0.17-0.85)	0.37 (0.12-1.20)
AS-AQ	DH-PP	0.32* (0.12-0.80)	0.12* (0.02-0.52)	0.61 (0.22-1.66)	0.28 (0.05-1.36)

Treatment outcome on day 28 for studies conducted in 2005-2007. OR (ITT), odds ratio in the case of the intention-to-treat (ITT) analysis, where the total number of patients enrolled was considered. OR (PP), odds ratio in the per-protocol (PP) analysis.

^1^Treatment taken as reference. Odds-ratios (OR, 95% confidence intervals in parentheses) were calculated for the pairs of ACT in each randomized study, except for the study in 2005 (AS-AQ vs AQ). Asterisks denote *P *< 0.05.

Based on the PCR-corrected proportions of ACPR on day 28, there was no statistical difference in the efficacy of AQ-SP and AS-MQ combinations (ITT: 92.5% AQ-SP vs 88.4% AS-MQ; OR, 1.62, 95% CI, 0.50-5.24). The decrease in parasitaemia was more rapid with AS-MQ than AQ-SP on day 2 (OR = 10.0, 95% CI, 4.2-23.6, *P *< 0.05). However, on day 3 (see Table 1), the proportions of parasite clearance were similar (95.7% vs 86.6%; *P *= 0.13). Moreover, more than 90% of patients cleared their parasitaemia on day 3, except for the AQ-SP combination and AQ monotherapy.

Treatment failure occurred in one out of 61 patients (1.7%, PCR-uncorrected LPF) treated with AS-MQ on day 28. Failure was observed in five additional patients (one LPF and four LCF, PCR-uncorrected; one lost-to-follow-up) between day 29 and day 42. Vomiting in children treated with AS-MQ occurred more frequently than in the AQ-SP group, on day 1 (10.3% vs 1.5%), day 2 (10.8% vs 0) and day 3 (3.3% vs 1.6). Significant difference was observed on day 1 and 2 (*P *< 0.05). Three patients were excluded due to repeated vomiting associated with AS-MQ administration.

### Regression analyses

The logistic regression on pooled individual patient data (PCR-uncorrected) comparing the efficacy of ACT to that of AQ-SP showed that the efficacy of AQ-SP is not statistically different from that of AS-AQ, AS-MQ, AS-SP, and DH-PP on day 14 (Table 3). However, the efficacy of AS-CD was significantly lower (*P *< 0.05) than that of AQ-SP, at both endpoints on day 14 and day 28. AM-LM and DH-PP were significantly more effective than AQ-SP on day 28.

**Table 3:** Comparison of pooled PCR-uncorrected proportions of adequate clinical and parasitological response between AQ-SP and ACT.

ACT	Odds-ratio (95% CI), as compared to AQ-SP			
	
	day 2	day 3	day 14	day 28
AM-LM	25.6 (8.21-79.5)*	ND	2.81 (0.28-27.7)	6.32 (1.35-29.6)*
AS-AQ	26.4 (12.4-56.4)*	16.0 (3.35-76.1)*	0.72 (0.20-2.62)	0.80 (0.40-1.61)
AS-CD	34.7 (11.2-107)*	ND	0.26 (0.07-0.96)*	0.35 (0.16-0.75)*
AS-MQ	11.4 (4.76-27.1)*	3.26 (0.84-12.7)	0.36 (0.10-1.41)	1.45 (0.57-3.71)
AS-SP	18.9 (8.64-41.2)*	5.30 (1.57-17.9)*	0.91 (0.22-3.64)	1.34 (0.60-2.93)
DH-PP	37.4 (12.2-115)*	ND	0.80 (0.18-3.50)	9.16 (2.0-42.5)*

Patients who were excluded or lost-to-follow-up were not included in the analysis. Data from a total of 709 patients were analysed, with treatment as a single covariate. Asterisks denote *P *< 0.05. ND (not done) denotes infinite OR due to 100% ACPR. On day 7, 100% ACPR was observed with all bitherapies. ACT, artemisinin-based combination therapies; AS-AQ, artesunate-amodiaquine; AS-MQ, artesunate-mefloquine; AS-SP, artesunate-sulphadoxine-pyrimethamine; AM-LM, artemether-lumefantrine; AS-CD, artesunate-chlorproguanil-dapsone; DH-PP, dihydroartemisinin-piperaquine; AQ-SP, amodiaquine-sulphadoxine-pyrimethamine.

### Multi-treatment random-effects meta-analysis

**Figure 3:**
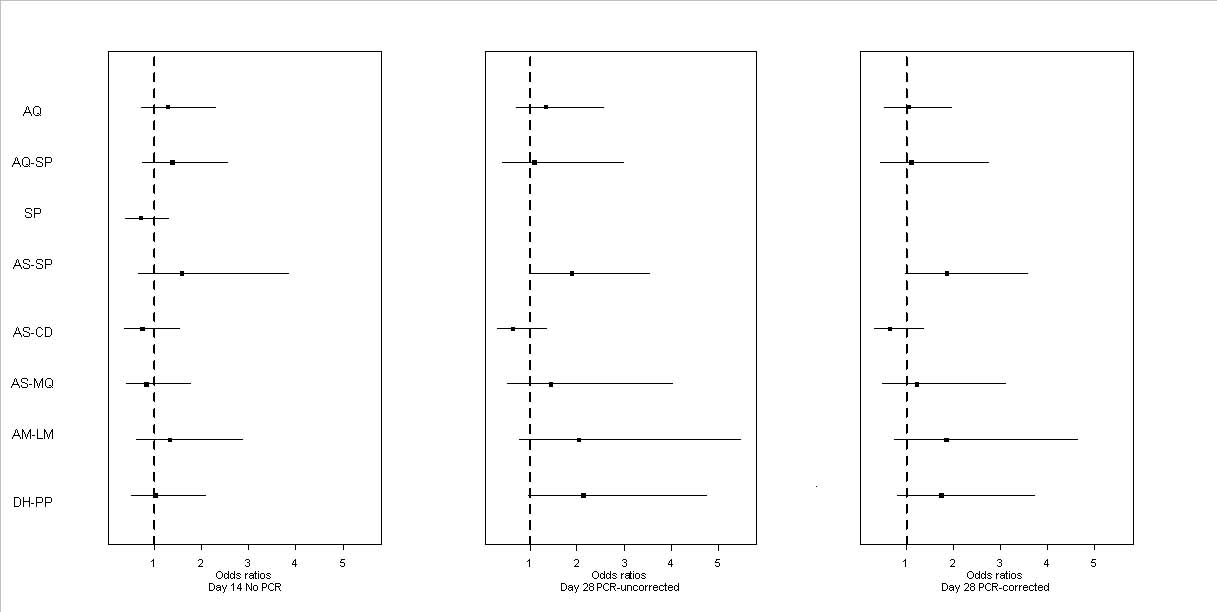
**Random-effects meta-analysis of treatment efficacy**. Posterior mean with 95% CI for odds ratios of each combination treatment with respect to AS-AQ (dotted line) for ACPR on day 14, and day 28 PCR uncorrected and corrected. Intention-to-treat analysis with individual patient data.

The results of the multi-treatment Bayesian random-effects meta-analysis based on individual data of children are shown in Figure 3. Posterior OR for each treatment was plotted. There was no significant difference in efficacy between AS-AQ and AM-LM: day 14 OR = 1.33 (95% CI, 0.62-2.88); day 28 PCR-uncorrected OR = 2.04 (0.76-5.47); day 28 PCR-corrected OR = 1.84 (0.73-4.66). The same conclusion holds for AS-CD, AS-MQ, AQ-SP, DH-PP, AS-SP, and AQ, on day 14 and on day 28, before and after PCR correction.

## Discussion

The present work concerned a global analysis of a series of randomized studies of anti-malarial treatment efficacy conducted in Cameroon between 2003 and 2007. Following comparison between arms within each study, a multi-treatment Bayesian random-effects meta-analysis of the binary outcome, ACPR/failure as a marker of efficacy, was carried out both on day 14 and day 28. The latter used PCR-uncorrected and PCR-corrected data. This global approach increased the power for detecting differences between treatments, while controlling the type-1 error.

Anti-malarials were AQ and SP monotherapies, their combination AQ-SP, and new drugs included in ACT. AQ monotherapy is still effective in Cameroon but should be protected with artesunate (or SP) to delay the emergence of resistance. The current trend in Africa is to reserve SP for the intermittent preventive treatment in pregnant women [15]. During the transition period before the actual implementation of the new drug policy based on ACT, AQ-SP combination has been proposed by some malaria experts to be an effective, alternative non-ACT combination [3]. The results showed that AQ-SP combination was more effective than AQ and SP monotherapies, in agreement with our earlier randomized study performed at another sentinel site and studies conducted elsewhere in Africa and Asia [5,16]. AQ-SP was as effective as AS-AQ combination, as already shown in a meta-analysis in Africa [17]. The advantages of AQ-SP combination include its high efficacy, good tolerance, suitability for young children, immediate availability of both drugs in many areas in Africa, and relatively low price of the generic drugs. Therefore, this non-ACT would have been a useful alternative during the transition period towards the full implementation of ACT to mutually protect AQ and SP in African countries where these two drugs are still effective.

In Cameroon, AS-AQ and AM-LM have being used nationwide since 2007 although AM-LM is relatively less prescribed due to its low supply in the public sector. The present study indicates that AS-AQ is well-tolerated and highly effective, confirming the results of an earlier multicentric study conducted in Africa [18]. Current concerns for the use of AS-AQ in Cameroon include the high number of individual non-coformulated AS and AQ tablets and the common perception that AQ intake provokes excessive fatigue and, in some patients, pruritus. The minor, transient side effects of AQ may lead to poor compliance and subsequent decline in AQ efficacy. The results of the present study highlighted a non-significant difference between AM-LM and AS-AQ. AM-LM is highly effective when the twice daily doses (total of six doses) are administered under supervision. As in the case of AS-AQ currently employed in Cameroon, there are concerns that six doses of AM-LM over three days may reduce compliance. Relatively few numbers of patients complained of physical fatigue during AM-LM treatment.

The forms of ACT, *i.e*. AS-SP, AS-CD, AS-MQ, DH-PP, that require once daily dose for three days, did not show a significant difference with AS-AQ. Previous studies have shown that AS-SP is a highly effective ACT [19]. However, in some African countries, this drug combination is not recommendable due to an increasing prevalence of antifolate resistance. The relatively higher number of reinfections observed between day 14 and day 28 in the AS-CD arms may partially be explained by the shorter elimination half-life of CD, compared with that of SP. Dapsone and chlorproguanil are antifolates that share similar chemical structures as sulphadoxine and pyrimethamine, respectively, and share the same mode of action. Although CD has been shown to be more effective than SP in several African countries, the development of AS-CD will not be further pursued by the drug manufacturer due to the rare, but severe, haematological adverse effects associated with dapsone [20-22].

AS-MQ combination has been widely used in some Southeast Asian countries to treat multidrug-resistant *P. falciparum *infections for more than a decade [23]. Its efficacy remains very high in Asia although some recent studies have suggested a possible decline in its efficacy [24-26]. In many parts of Africa, MQ, alone or in combination with SP, has rarely been used by the local populations. Initial studies of AS-MQ combination conducted in children aged > 5 years old and adults in Africa suggested its high efficacy (98-100% cure rate) and good tolerance [27,28]. The present study in children less than five years of age confirms the high efficacy of AS-MQ combination, although the corresponding frequency of vomiting seems to be in contradiction with those of previous studies, which involved sequential or simultaneous doses [27,29].

Piperaquine, an 'old' bisquinoline synthesized in the 1960s and used extensively in China, has been found to be a suitable partner of dihydroartemisinin [30]. Recent studies conducted in Asia have shown its high efficacy, safety, and good tolerance [31-33]. The results of the present study confirm its high efficacy and safety in malaria-infected African children. DH-PP may be a low-cost, effective alternative. Before piperaquine, in combination with dihydroartemisinin, is introduced at the regional level in Africa, its industrial production needs to conform to Good Manufacturing Practice standard [30].

The results of these studies on the efficacy of AS-AQ and AM-LM are in agreement with those conducted elsewhere in Africa [1]. The various forms of ACT that have not been extensively evaluated elsewhere, namely AS-MQ and DH-PP, are probably just as effective in other African countries. The choice of either AS-AQ or AM-LM for the treatment of uncomplicated malaria has led to some confusion among prescriptors, drug suppliers, and patients themselves in Cameroon. For a more rational drug distribution, an urgent measure is required for a clearer anti-malarial drug policy, defining clinical conditions in which alternative forms of ACT may be prescribed. Other effective forms of ACT (AS-SP, AS-CD, AS-MQ, DH-PP) may be more promising in terms of compliance. The possible role of AS-SP in combating drug-resistant *P. falciparum *in Central Africa is not well defined at present. In countries where SP is largely employed for intermittent preventive treatment in pregnant women, it may not be advisable to use AS-SP for malaria treatment of the general population. Further studies are required to evaluate the optimal dosing of AS-MQ for African children. At present, it is probably too early to recommend AS-MQ in Africa as an alternative to other existing forms of ACT, which are better tolerated than AS-MQ. There are other novel forms of ACT, including artesunate-pyronaridine and artesunate-atovaquone-proguanil, that have not been evaluated in the present study. Clinical efficacy and tolerance of these combinations need to be evaluated and compared with those of AS-AQ and AM-LM in Central Africa.

## Conflict of interest statement

The authors declare that they have no competing interests.

## Authors' contributions

SWY developed the analysis plan and carried out both the statistical analyses and software implementations under the close supervision of HG and JCT. All participated in the interpretation of data. RT was responsible for checking the data and performing molecular techniques. VFN supervised the enrolment and follow-up of patients and participated in data entry and collection. GS designed the studies and assisted with data interpretation. LKB was responsible for overall scientific management and drafted the manuscript. All authors participated in the preparation of the report and approved the final version.

## Supplementary Material

Additional file 1 **Table S1: Pre-treatment clinical and laboratory characteristics of enrolled children who completed the 14-day or 28-day follow-up**. ^1 ^Patients were followed-up for 14 days in studies conducted in 2003 and for 28 days in studies performed in 2005-2007. Patients assigned to artesunate-mefloquine group were followed for 42 days. AQ, amodiaquine; SP, sulphadoxine-pyrimethamine; AS, artesunate; MQ, mefloquine; AM, artemether; LM, lumefantrine; CD, chlorproguanil-dapsone; DH, dihydroartemisinin; PP, piperaquine. ^2 ^Number of patients enrolled (number of patients analyzed, with complete 14- [in 2003] or 28-day [in 2005-2007] follow-up, in parentheses). ^3 ^The numbers of children aged > 60 months old (and/or adults for Maroua) are 18/57 in Garoua 2003 AQ, 16/58 in Garoua 2003 SP, 27/58 in Garoua 2003 AQ-SP, and 18/64 (28.1%) in Maroua (none at other study sites). Garoua and Maroua are situated in northern Cameroon where malaria transmission is seasonal. ^4 ^The following number of patients had > 200,000 asexual parasites/μL of blood: 2 (1 in AQ group and 1 in SP group) in Yaoundé 2003; 5 (2 in AQ group and 3 in SP group) in Bertoua 2003; 3 (2 in AQ group and 1 in AQ-SP group) in Garoua 2003; 10 (5 in AQ group, 4 in AS-AQ group, and 1 in AS-SP group) in Yaoundé 2005; 1 in Maroua; 4 (2 in AQ-SP group, 2 in AS-MQ group) in Yaoundé 2006a; 5 (4 in AS-AQ group, and 1 in AM-LM group) in Yaoundé 2006b; 7 (5 in AS-SP group and 2 in AS-CD group) in Yaoundé 2007a; and 11 (5 in DH-PP group and 6 in AS-AQ group) in Yaoundé 2007b. Click here for file
