# Supplementary material for: Efficacy of non-artemisinin- and artemisinin-based combination therapies for uncomplicated falciparum malaria in Cameroon
Source: Malar J. 2010 Feb 19;9:56. doi: 10.1186/1475-2875-9-56 (PMC2834703; doi:10.1186/1475-2875-9-56)
Supplement: Additional file 1 — Table S1: Pre-treatment clinical and laboratory characteristics of enrolled children who completed the 14-day or 28-day follow-up. 1 Patients were followed-up for 14 days in studies conducted in 2003 and for 28 days in studies performed in 2005-2007. Patients assigned to artesunate-mefloquine group were followed for 42 days. AQ, amodiaquine; SP, sulphadoxine-pyrimethamine; AS, artesunate; MQ, mefloquine; AM, artemether; LM, lumefantrine; CD, chlorproguanil-dapsone; DH, dihydroartemisinin; PP, piperaquine. 2 Number of patients enrolled (number of patients analyzed, with complete 14- [in 2003] or 28-day [in 2005-2007] follow-up, in parentheses). 3 The numbers of children aged > 60 months old (and/or adults for Maroua) are 18/57 in Garoua 2003 AQ, 16/58 in Garoua 2003 SP, 27/58 in Garoua 2003 AQ-SP, and 18/64 (28.1%) in Maroua (none at other study sites). Garoua and Maroua are situated in northern Cameroon where malaria transmission is seasonal. 4 The following number of patients had > 200,000 asexual parasites/μL of blood: 2 (1 in AQ group and 1 in SP group) in Yaoundé 2003; 5 (2 in AQ group and 3 in SP group) in Bertoua 2003; 3 (2 in AQ group and 1 in AQ-SP group) in Garoua 2003; 10 (5 in AQ group, 4 in AS-AQ group, and 1 in AS-SP group) in Yaoundé 2005; 1 in Maroua; 4 (2 in AQ-SP group, 2 in AS-MQ group) in Yaoundé 2006a; 5 (4 in AS-AQ group, and 1 in AM-LM group) in Yaoundé 2006b; 7 (5 in AS-SP group and 2 in AS-CD group) in Yaoundé 2007a; and 11 (5 in DH-PP group and 6 in AS-AQ group) in Yaoundé 2007b. [file 1475-2875-9-56-S1.DOC]

Additional File S1. Pre-treatment clinical and laboratory characteristics of enrolled children who completed the 14-day or 28-day follow-up.

| Study site (year, treatment group)^1^ | n^2^ | age  mean ± SD  (range)^3^  months | weight  mean±SD  (range)  kg | sex ratio  (M:F) | haematocrit  mean ± SD  (range)  % | parasitaemia  geometric  mean,  range^4^  per µL of  blood | rectal  temperature  mean ± SD  (range)  °C | proportion  of self-  medication,  anti-malarials  % | proportion  of self-  medication,  anti-pyretics  % |
| --- | --- | --- | --- | --- | --- | --- | --- | --- | --- |
| Yaoundé  2003 AQ | 64 (63) | 28.2 ± 19.2  (3–60) | 12.4 ± 4.1  (6–20) | 0.90 | 27.4 ± 5.7  (16–39) | 28,000  2,100–220,000 | 38.8 ± 0.8  (38.0–41.0) | 52.4 | 81.0 |
| Yaoundé  2003 SP | 61 (61) | 27.1 ± 16.1  (4–58) | 12.8 ± 4.1  (6–22) | 1.54 | 27.8 ± 6.2  (15–40) | 31,000  2,900–260,000 | 38.9 ± 0.8  (38.0–41.0) | 34.4 | 62.3 |
| Yaoundé  2003  AQ-SP | 62 (59) | 25.2 ± 15.6  (6–58) | 12.3 ± 3.8  (6–23) | 1.27 | 27.2 ± 5.2  (14–39) | 30,000  6,400–180,000 | 38.8 ± 0.8  (38.0–41.0) | 37.3 | 69.5 |
| Bertoua  2003 AQ | 58 (54) | 26.7 ± 15.8  (6–58) | 11.7 ± 2.9  (8–19) | 1.07 | 27.9 ± 5.2  (16–41) | 20,000  2,000–250,000 | 38.7 ± 0.6  (38.0–41.0) | 45.9 | 89.2 |
| Bertoua  2003 SP | 57 (53) | 26.9 ± 15.9  (6–58) | 12.1 ± 3.6  (7.5–21) | 0.80 | 27.7 ± 4.6  (18–37) | 25,100  2,830–200,000 | 38.8 ± 0.7  (38.0–40.0) | 55.6 | 88.9 |
| Bertoua  2003AQ-SP | 61 (56) | 33.5 ± 17.4  (7–60) | 12.8 ± 4.1  (7–21) | 0.96 | 27.6 ± 6.0  (12–38) | 22,700  2,150–184,000 | 38.9 ± 0.7  (38.0–40.0) | 67.5 | 92.5 |
| Garoua  2003 AQ | 58 (57) | 52.3 ± 29.4  (8–106) | 14.9 ± 5.2  (7–28) | 1.37 | 30.8 ± 4.1  (20–38) | 30,500  3,000–300,000 | 39.2 ± 0.7  (38.0–41.3) | 56.8 | 100 |
| Garoua  2003 SP | 61 (58) | 47.8 ± 26.5  (10–108) | 12.8 ± 4.4  (7–25) | 0.93 | 30.6 ± 5.5  (20–40) | 39,000  3,300–194,000 | 39.1 ± 0.7  (38.0–40.8) | 65.9 | 90.2 |
| Garoua 2003 AQ-SP | 60 (58) | 58.9 ± 29.2  (8–106) | 17.1 ± 5.6  (7.4–30) | 0.93 | 30.1 ± 6.4  (15–41) | 34,700  2,300–237,000 | 39.2 ± 0.8  (38.0–40.7) | 63.3 | 93.9 |
| Yaoundé  2005 AQ | 64 (59) | 28.5 ± 17.2  (6–58) | 13 ± 4.2  (6.4–22) | 0.94 | 27.3 ± 4.8  (15–36) | 30,300  2,100–250,000 | 39.0 ± 0.75  (38.0–40.9) | 31.2 | 76.5 |
| Yaoundé  2005 AS-AQ | 60 (54) | 28.9 ± 17.4  (6–58) | 13.2 ± 4.2  (7–24) | 0.93 | 28.3 ± 6.1  (15–40) | 32,900  2,000–255,000 | 38.9 ± 0.63  (38.0–40.8) | 38.3 | 76.6 |
| Yaoundé  2005 AS-SP | 61 (57) | 33.4 ± 18.8  (6–60) | 13.5 ± 4.35  (2–27) | 1.03 | 27.4 ± 5.5  (15–38) | 43,000  2,050–  220,000 | 39.1 ± 0.83  (38.0–41.0) | 31.1 | 70.5 |
| Maroua  2005 AS-  AQ | 64 (58) | 153 ± 18.2  (7–660) | 29.3 ± 20.5  (7–96) | 0.56 | 29.6 ± 0.82  (16–48) | 26,200  2,020–  360,000 | 39.0 ± 0.72  (38.0–41.0) | 28.1 | 46.9 |
| Yaoundé  2006a  AQ-SP | 67 (62) | 29.9 ± 17.5  (6–60) | 13.3 ± 4.1  (6–25) | 0.90 | 26.6 ± 4.6  (17–38) | 40,100  2,050–  320,000 | 38.9 ± 0.64  (38.0–40.9) | 16.4 | 62.7 |
| Yaoundé  2006a  AS-MQ | 69 (61) | 28.8 ± 16.0  (7–60) | 12.3 ± 3.4  (7–22) | 1.54 | 25.8 ± 5.5  (15–40) | 34,600  2,000–  300,000 | 38.9 ± 0.59  (38.0–40.4) | 13.0 | 68.1 |
| Yaoundé 2006b AS-AQ | 62 (57) | 30.1 ± 17.6  (6–60) | 13 ± 4.5  (6.5–25) | 0.77 | 29.2 ± 4.9  (20–42) | 30,300  2,000–512,000 | 39.0 ± 0.71  (38.0–40.7) | 11.3 | 46.8 |
| Yaoundé 2006b AM-LM | 61 (60) | 28.9 ± 16.2  (6–59) | 12.7 ± 3.8  (6–21) | 0.97 | 28.2 ± 5.2  (15–38) | 33,000  2,000–247,000 | 38.9 ± 0.73  (38.0–40.8) | 8.2 | 57.4 |
| Yaoundé 2007a AS-SP | 85 (79) | 33.8 ± 17.7  (6–60) | 14.5 ± 3.1  (7–40) | 1.4 | 28.4 ± 4.2  (20–39) | 30,600  2,010–366,000 | 38.9 ± 0.70  (38.0–41.0) | 4.7 | 62.4 |
| Yaoundé 2007a AS-CD | 83 (71) | 36.8 ± 17.4  (4–60) | 14.8 ± 4.1  (6.5–24) | 1.2 | 28.8 ± 5.3  (16–40) | 33,900  2,010–218,000 | 38.9 ± 0.66  (38.0–40.5) | 6.0 | 56.7 |
| Yaoundé 2007b DH-PP | 91 (86) | 29.3 ± 17.8  (7–60) | 13.1 ± 1.2  (5–25) | 0.75 | 28.4 ± 4.6  (18-38) | 40,170  2,050–310,857 | 38.9 ± 0.79  (38.0–40.1) | 3.3 | 68.1 |
| Yaoundé 2007b AS-AQ | 92 (88) | 32.2 ± 17.5  (6–60) | 13.5 ± 4.4  (3–24) | 1.1 | 28.4 ± 4.8  (16–40) | 45,034  2,100–500,000 | 38.9 ± 0.75  38.0–41.9 | 4.3 | 58.7 |
